# Recent advances in understanding corticotroph pituitary tumor initiation and progression

**DOI:** 10.12688/f1000research.14789.1

**Published:** 2018-08-29

**Authors:** Ulrich Renner, Denis Ciato, Günter K. Stalla

**Affiliations:** 1 Max Planck Institute of Psychiatry, Clinical Neuroendocrinology Group, Munich, Germany

**Keywords:** corticotroph tumor, USP8 mutation, progression, corticotroph carcinoma

## Abstract

Cushing’s disease is the most frequent form of hypercortisolism and is caused by hypophyseal corticotroph adenomas secreting excessive amounts of adrenocorticotropic hormone. Most of the tumors develop sporadically and only a limited number of corticotroph adenomas have been found to be associated with different neuroendocrine syndromes or with familial isolated pituitary adenomas. The pathogenic mechanisms of corticotroph adenomas are largely unknown, but the discovered aberrant chaperoning activity of heat shock protein 90 on the one hand and the presence of ubiquitin-specific protease 8 mutations on the other hand partially explained the causes of their development. Corticotroph tumors arise initially as benign microadenomas but with time form invasively growing aggressive macroadenomas which can switch to corticotroph carcinomas in extremely rare cases. The mechanisms through which corticotroph tumors escape from glucocorticoid negative feedback are still poorly understood, as are the processes that trigger the progression of benign corticotroph adenomas toward aggressive and malignant phenotypes. This review summarizes recent findings regarding initiation and progression of corticotroph pituitary tumors.

## Introduction

Corticotroph adenomas (CAs) derive from normal corticotroph cells of the anterior pituitary and account for about 4% to 8% of all clinically hormone-active anterior pituitary tumor types ^1^. Including clinically inactive silent corticotroph tumors, CAs encompass up to 15% of all pituitary tumors ^2^. The prevalence for hormone-active CAs is about 40 cases per million and the incidence ranges from 1.2 to 2.4 per million per year. In adults, CAs are diagnosed mainly in the fourth to sixth decade of life and are about three times more prevalent in women ^1^. No gender preference has been observed in CAs in children, in whom these tumors are rare (<10% of all CAs) ^3^.

CAs have rarely been described with hereditary background, and in the vast majority of sporadic CAs, the genetic background is still largely unknown ^4^. Whereas silent CAs are immunopositive for adrenocorticotropic hormone (ACTH), but do not manifest biochemical or clinical hypercortisolism ^5^, the majority of clinically active CAs secrete excessive amounts of ACTH, causing chronic hypercortisolism due to lack of hypothalamus–pituitary–adrenal (HPA) axis feedback regulation at the level of the CA, leading to manifestations of the clinical features of Cushing’s disease ^1^. Normally, there is a delay of several years between the onset of the disease and its diagnosis because early symptoms of Cushing’s disease are rather unspecific. At the time of diagnosis, about 90% of the patients present with corticotroph microadenomas less than 1 cm in diameter; in some cases, these tumors are hardly detectable with magnetic resonance imaging ^1^. Transsphenoidal surgery is generally the initial treatment of choice but is not always successful. In the case of incomplete resection (for example, if the tumor is critically located in proximity to the optical nerves), the tumor might expand and become more aggressive over time, requiring repeated surgery, radiotherapy, pharmaceutical therapy, or adrenalectomy ^1^. In rare cases, CAs finally transform to metastasizing corticotroph carcinomas for which temozolomide treatment has recently been established with some success in single cases ^6, 7^.

## Genetics of hereditary corticotroph adenomas

Less than 5% of all CAs are familial adenomas representing rare tumor manifestations in various hereditary endocrine syndromes—multiple endocrine neoplasia 1 (MEN1), MEN4, Carney complex (CNC), and DICER1 syndrome—or in familial isolated pituitary adenomas (FIPAs) ^4, 8^. Patients with MEN1, which is caused by a mutation of the *MEN1* gene encoding the tumor suppressor menin, mainly manifest prolactinomas and somatotropinomas and only about 5% of their totality is represented by corticotroph tumors ^8, 9^. So far, a single case of CA has been described in MEN4, in which the cell cycle regulator p27 ^Kip1^ is impaired by inactivating mutations of the *CDKN1B* gene ^10^. In CNC, caused by inactivating germline mutations of the *PRKAR1A* gene which encodes the type 1α regulatory subunit of protein kinase A, one case of CA has been reported ^11^. In DICER1 syndrome, which is associated with the development of different types of blastomas during embryogenesis, 2% of the affected patients develop corticotroph blastomas as the only pituitary blastoma manifestation ^12, 13^. Patients with DICER1 syndrome bear an autosomal dominant mutation of the *DICER1* gene which encodes the DICER protein, a microRNA (miR) processing endoribonuclease. This suggests that a disturbed production of miRs and thus an altered regulation of their target mRNAs may be responsible for the development of the corticotroph blastomas. However, it is not yet known which miRs are affected and how they induce corticotroph tumor development or why only a small subset of affected patients develops corticotroph blastomas ^12, 13^.

Hereditary disorders, in which the patients manifest only pituitary tumors, are designated FIPAs. About 20% of all patients with FIPA bear mutations of the aryl hydrocarbon receptor-interacting protein (AIP), whereas in the majority of FIPAs, the underlying mutations are not known. CAs account for about 5% of all pituitary tumors in FIPAs ^14^.

In several studies with big cohorts of patients with sporadic pituitary adenoma, only three cases of pathogenetic AIP mutations and three cases of menin mutations have been found in sporadic corticotropinomas, suggesting that mutations of genes causing hereditary corticotroph tumors play only a minor role in sporadic CA development ^4, 15, 16^.

## Genetic and epigenetic modifications in sporadic corticotroph adenomas

Most of the CAs (>95%) are sporadic tumors of monoclonal origin ^17, 18^. Loss of heterozygosity in combination with different mutations leading to loss of tumor suppressor genes or activation of oncogenes (or both) may play a role in the initiation of sporadic CAs ^19^.

Despite considerable progress during the last decade, sufficient knowledge about the genetic background of CAs is still missing. Important progress has been made with the recent detection of recurrent gain-of-function mutations in the gene encoding a protein called ubiquitin-specific protease 8 (USP8) in about 20% to 60% of CAs in adult patients ^20– 22^. As the mutation was not found in any other type of pituitary tumors, it is evident that the mutation is specific for CAs ^23^. USP8 is involved in the ubiquitination/deubiquitination process, which is a modality of post-translational protein modification ^24, 25^. In this complex process, specific proteins are ubiquitinated and then degraded by the lysosome or are rescued from degradation by deubiquitination. USP8 is an enzyme that mediates the deubiquitination of target proteins, and described mutations in CAs lead to an increased activity of the protein. CAs with mutated USP8 exhibit more stable or higher levels of proteins whose activities are regulated by ubiquitination/deubiquitination ^20, 21^.

Multiple proteins are known to be regulated at least in part by ubiquitination/deubiquitination, and in principal all USP8 regulated proteins, if expressed in CAs, could be affected by USP8 mutations ^24, 25^. In this context, two candidate proteins that might play a pathogenic role and be a pharmacological target in CAs with mutated USP8 have already been identified: the epidermal growth factor receptor (EGFR) ^20, 21^ and the somatostatin receptor type 5 (SSTR5) ^26^. The tyrosine kinase receptor EGFR is expressed in corticotroph cells and mediates the ACTH-stimulating effect of the epidermal growth factor (EGF) and transforming growth factor-alpha (TGF-α) ^27– 29^. In CAs with USP8 mutations and thus enhanced deubiquitination, EGFR is stabilized and its expression is increased. Therefore, the ACTH-stimulating activity of EGF/TGF-α is elevated and may contribute to the excessive ACTH production. Indeed, comparative studies between CAs with and without mutated USP8 have already shown that the expression and activity of the EGFR are enhanced in CAs with USP8 mutations, ^20, 21^ although this could not be confirmed in another study ^26^. In the case of SSTR5, USP8 mutations would lead to an increased expression and activity of this receptor and suggest that CAs with mutated USP8 may respond better to the treatment with pasireotide, a somatostatin analogue that suppresses CA growth and ACTH secretion through SSTR5 ^26, 30^. In addition, a Cushing’s disease–relevant protein, whose expression or function or both are regulated at least in part by ubiquitination/deubiquitination (and thus may be affected by USP8 mutations), is the glucocorticoid receptor (GR) ^31^. The latter regulates the responsiveness to glucocorticoids, resistance to which is a typical feature of Cushing’s disease. Whether a reduced sensitivity to glucocorticoids by CAs is related to the mutational status of USP8 still needs to be clarified. Interestingly, a recent article has shown evidence that in normal rat corticotroph cells the proopiomelanocortin (POMC) peptide is directly degraded by ubiquitination and therefore that the treatment of rat corticotropes with the polyubiquitination inhibitor K48R enhanced ACTH production ^32^. Whether this is also relevant in human corticotroph tumor cells has yet to be proven. Comparative investigations have demonstrated that CAs with mutated USP8 are smaller and that the prevalence for USP8 mutations is higher in women ^20, 21, 33^. In a recent study, a lower incidence of USP8 mutations was found in children with Cushing’s disease compared with adults ^33, 34^. Whereas the prevalence of USP8 mutations was reduced in Crooke’s cell adenomas ^26^, the proportion of mutant USP8 in so-called Nelson’s tumors (see the “Corticotroph pituitary tumor types” section) was identical to that in corticotropinomas in general ^35^. Patients with USP8 mutations were diagnosed significantly earlier and had higher preoperative 24-hour urinary-free cortisol levels ^36^. After surgery, recurrences were more abundant and appeared significantly earlier in patients with USP8 mutant corticotropinomas ^36^. In contrast to ACTH-secreting CAs, no USP8 mutations have been detected in a small number (n = 13) of silent CAs studied ^33^ but this has to be confirmed in a bigger cohort of this tumor type. Interestingly, no mutant USP8 has been found in tumors from a big cohort of dogs with Cushing’s disease, suggesting that the mutation is primate-specific ^37^.

So far, apart from mutated USP8, no other recurrent mutations have been found in CAs ^4, 23, 38^. Thus, more than half of the CA cases may have a variable genetic background and may be associated with mutations of different single genes or may be caused by epigenetic mechanisms (see below). So far, several mutations of different genes have been described in CAs, which mainly affected cell cycle–regulating proteins, signal transduction proteins, oncogenes, and tumor suppressors ^4, 23, 38, 39^. Very recently, loss-of-function mutations of the Cdk5 and ABL enzyme substrate 1 ( *CABLES1*) gene were found in four patients from a cohort of 146 pediatric and 35 adult patients with Cushing’s disease: two in children and two in young adults ^40^. The mutated CABLES1 protein lost its inhibitory action on corticotropinoma cell growth, which may explain why all affected patients had corticotroph macroadenomas ^40^.

Given that mutations play a tumorigenic role in only a part of the corticotropinomas, it is thought that epigenetic changes such as DNA methylation, histone methylation/acetylation, and miR are of considerable impact for corticotropinoma formation ^41, 42^. Most studies on this subject have been performed in unselected cohorts of different types of pituitary adenomas; among them, corticotropinomas and epigenetic changes of different factors have been detected in subsets of the adenomas. Corticotropinoma-specific studies on histone deacetylases have demonstrated that a loss of HDAC2 might play a role in inducing glucocorticoid resistance ^43^, and treatment with histone deacetylase inhibitors reduced survival and ACTH secretion in corticotroph tumor cells ^44^. Many of the epigenetic changes caused by DNA methylation or histone methylation/acetylation have direct or indirect effects on the expression of the tumor suppressors p53 and retinoblastoma (Rb) protein, suggesting that these factors are prominent targets of epigenetic changes ^41, 42^. A case report of loss of Rb expression in a corticotroph carcinoma ^45^ supports the concept that this tumor suppressor plays a role in corticotropinoma development but apparently it is involved in tumor progression rather than in adenoma initiation.

In regard to miRs, which bind to specific messenger RNAs and block their translation into corresponding proteins, several entities have been reported to be either over- or under-expressed in pituitary adenomas in general ^46– 48^ or specifically in CAs ^49– 51^. For instance, invasive characteristics of pituitary tumors were found to be associated with the over-expression of the miR-106b~25 cluster consisting of miR-25, miR-93, and miR-106b ^50^. Moreover, aggressive corticotroph Crooke’s cell adenomas had a higher expression of miR-106b~25 than other corticotropinomas, suggesting that this miR cluster is also associated with corticotroph tumor aggressiveness ^50^. In another study about miR expression specifically in corticotropinomas, patients presenting reduced miR-141 levels showed a better chance of remission, whereas the reduced expression of other miRs (miR-15a, miR-16, miR-21, miR-141, miR-143, miR-145, miR-150, and let-7a) had no association with corticotropinoma phenotype or clinical parameters of the affected patients ^49^. The targets and pathological consequences of miRs in CAs have been identified in only a few cases ^48, 51^, and much more work has to be done to identify the pathological mechanisms of aberrant miR expression.

## Glucocorticoid resistance in corticotroph adenomas

In normal corticotroph cells, ACTH production and release are under negative feedback control of glucocorticoids and are well balanced. If needed, the ACTH and thus glucocorticoid production can be transiently adapted to specific physiological or pathophysiological conditions (stress, infections, inflammation, and so on) ^52^. Corticotroph tumor cells produce excessive amounts of ACTH despite strongly elevated glucocorticoid serum levels, indicating that the negative glucocorticoid feedback is impaired. It is still not clear why corticotroph tumor cells are resistant to glucocorticoids. Inactivating mutations of the GR encoding nuclear receptor subfamily 3 group C member 1 ( *NR3C1*) gene are rare, and as corticotroph tumor cells show mostly enhanced GR levels, the missing response to glucocorticoids cannot be explained by GR downregulation ^23, 39, 53, 54^. Thus, an alternate impairment of the function of GR must be responsible for the glucocorticoid resistance, and two candidates—the testicular orphan nuclear receptor 4 (TR4) and heat shock protein 90 (HSP90)—are discussed to play important roles in GR dysfunction.

TR4 is a nuclear receptor which can both activate and repress transcription in different types of organs. TR4 is overexpressed in CAs and corticotroph tumor cell lines and is able to activate the ACTH encoding *POMC* gene by binding to its promoter ^55, 56^. The activation is further enhanced if TR4 is phosphorylated through the mitogen-activated protein kinase/extracellular signal-regulated kinase (MAPK/ERK) pathway. In nude mice harboring corticotroph tumors, TR4 overexpression stimulated ACTH secretion and tumor cell growth ^55^. It was shown that TR4 interacts with the GR and could overcome the negative regulation of GR on *POMC* transcription and ACTH secretion, suggesting that TR4 might promote resistance to negative glucocorticoid feedback ^55, 56^. Suppression of ERK-mediated TR4 phosphorylation by the ERK inhibitor MEK-162 in a murine model of corticotroph tumors reduced ACTH production and inhibited tumor growth, indicating that targeting TR4 action or suppression (or both) could be a novel therapy for Cushing’s disease ^57^.

HSP90 is a chaperone protein that stabilizes and regulates different proteins by inducing conformational changes. HSP90 interferes with the GR to facilitate ligand binding and receptor translocation to the nucleus and therefore plays an important role in the function of the GR ^58^. However, under certain conditions, HSP90 can impair GR function ^59, 60^. HSP90 expression is strongly enhanced in the anterior pituitary in patients with CA in comparison with the normal anterior pituitary, and it was shown that HSP90 overexpression restrains the release of mature GR from the chaperone, leading to partial glucocorticoid resistance ^61^. C-terminal inhibitors of HSP90 such as novobiocin and the herbal compound silibinin could induce the release of mature GR in corticotroph tumor cells *in vitro*, and *in vivo* silibinin was able to revert glucocorticoid resistance in a corticotroph tumor allograft mouse model, leading to a partial restoration of symptoms of the disease ^61^. This suggests that silibinin, a safe drug already used in humans for the treatment of liver disease and amanitin intoxication ^62^, may represent a novel therapeutic option for the treatment of Cushing’s disease and corresponding clinical studies are currently in preparation.

## Progression of corticotroph adenomas

### Corticotroph pituitary tumor types

According to the 2017 World Health Organization (WHO) classification of pituitary tumors, CAs are characterized by the expression of Tpit, a transcription factor specifically expressed in the ACTH-producing pituitary cell lineage. Histologically, hormone-secreting CAs are further subclassified into densely and sparsely granulated CAs and Crooke’s cell adenomas ^2^. Not classified as a specific entity in the 2017 WHO classification are Nelson’s tumors, the original designation of aggressively progressing corticotroph macroadenomas developing in 8% to 29% of patients, in which the chronic hypercortisolism had been treated by bilateral adrenalectomy ^63– 65^. It was thought that the removal of cortisol excess could play a causative role in the development of Nelson’s tumors. However, as CAs in adrenalectomized patients mostly remain stable or progress slowly and as the patients receive adequate cortisol replacement therapy, it is unlikely that the adrenalectomy-associated removal of excessive cortisol alone is responsible for the formation of Nelson’s tumors ^63– 65^. Nevertheless, owing to elevated proliferative index and enhanced invasive growth, Nelson’s tumors can evolve to carcinomas in rare cases ^66^. There is no evidence for a common specific genetic background leading to the formation of Nelson’s tumors, and in a recent study the proportion of USP8 mutations (45%) was found to be similar to that of corticotroph tumors in general ^35^. Thus, the mechanisms triggering the development of Nelson’s tumors are still unknown ^64, 65^.

Crooke’s cell adenomas are characterized by the replacement of cytoplasmic granules by hyaline material ^2^, a process that has been designated Crooke’s change. In a recent study, it was shown that depending on the degree of hypercortisolism, densely/sparsely corticotroph tumors show focal Crooke’s changes ^67^. This suggests that Crooke’s cell adenomas have no specific genetic/epigenetic background but derive from densely/sparsely CAs at a high hypercortisolemic state. This is supported by the observation that there is little difference in the proportion of USP8-mutated and non-mutated tumors between densely/sparsely granulated CAs and Crooke’s cell adenomas ^26^. At present, the underlying mechanisms triggering the development of Crooke’s cell adenomas are not known. In the 2017 WHO high-risk pituitary adenoma subclassification, Crooke’s cell adenomas are listed as an aggressive tumor type as these tumors have an elevated proliferative index and are already invasively growing at a microadenoma state ^2^. As Crooke’s cell adenomas are at risk to transform to corticotroph carcinomas, chemotherapeutic treatment of these adenomas is recommended to prevent the transformation ^68^. Silent CAs also belong to the aggressive pituitary tumor types in the new WHO classification. This tumor entity is immunopositive for Tpit and expresses different POMC precursor proteins but does not secrete ACTH; moreover, genes related to ACTH synthesis are differently expressed in comparison with ACTH-secreting CAs ^2, 69^. Thus, silent CAs clinically represent a member of the group of non-functioning pituitary adenomas (NFPAs), and according to recent studies, up to 20% of all NFPAs are silent CAs ^5^. There are several hypotheses and speculations about silent CAs and their inability to secrete ACTH. Silent CAs may not derive from normal anterior pituitary corticotroph cells but may originate from POMC-expressing cells of the pars intermedia and thus may have characteristics different from those of “normal” CAs ^69^. There is also electron microscope–based evidence that silent CAs have elevated lysosome numbers and show fusions of these lysosomes with secretory granules and this has led to speculation that ACTH is intracellularly destroyed before it can be secreted ^70^. Alternatively, the function or expression of POMC product–processing enzymes may be disturbed in silent CAs and indeed abnormalities of these prohormone convertases have been described ^71^. However, the processes responsible for silent CA development still need to be clarified in future studies. The observation that silent corticotropinomas probably have no mutated USP8 may indicate that silent CAs have a different genetic background compared with classic CAs ^33^. However, in some cases, silent CAs have spontaneously changed to secretory CAs and vice versa, suggesting that at least in some cases silent and secretory CAs may have the same origin ^72^. Like most NFPAs, silent CAs are diagnosed quite late when they have already formed invasively growing macroadenomas. At this state, complete surgical resection is not possible and further clinical management of these tumors is quite challenging; in rare cases, silent CAs finally transform to carcinomas ^73, 74^.

### Microadenomas, macroadenomas, and aggressive corticotroph tumors

At diagnosis, most of the ACTH-secreting CAs are microadenomas and in some cases are hardly visible by routine imaging techniques ^1^. When CAs have reached a critical size of more than 2 mm in diameter, oxygen transport to the tumor cells per diffusion will no longer be sufficient and will lead to tumor neovascularization through angiogenesis forced by intratumoral hypoxia ^75, 76^. The most important regulator of angiogenesis is the hypoxia-inducible factor-1 (HIF-1), which is inactive under normoxic conditions but active under hypoxia ^77^. Activated HIF-1 induces the production of multiple angiogenic factors—for example, vascular endothelial growth factor-A (VEGF-A), basic fibroblast growth factor, and platelet-derived growth factor—and suppresses the formation of anti-angiogenic components. This finally stimulates the proliferation of endothelial cells and pericytes, promotes the formation of tubular structures, and induces the targeted sprouting of the newly formed vessels into the hypoxic zones of the growing tumor ^77^. HIF-1 is a heterodimeric protein composed of two subunits: oxygen-regulated HIF-1α and constitutively expressed HIF-1β ^78^. Under normoxic conditions, HIF-1α has a very short half-life due to permanent degradation by ubiquitination and therefore little or no active HIF-1 is present. During hypoxia, the ubiquitination of HIF-1α is blocked and active HIF-1 is formed to induce the angiogenic processes ^78^. As the previously mentioned USP8 protein interferes with HIF-1α deubiquitination processes ^25, 79^, it would be interesting to study whether HIF-1 levels are different in corticotropinomas with and without mutated USP8 and whether corticotropinoma vascularization might be affected as in the case of the product of the *RWWD3* gene, RWD-domain-containing sumoylation enhancer (RSUME) ^80^. The latter is upregulated under hypoxia and overexpressed in pituitary tumors, including corticotropinomas ^81^. It has been shown that RSUME could stabilize HIF-1 and thus enhance VEGF-A secretion. Therefore, the knock-down of RSUME was associated with reduced production of HIF-1 and VEGF-A in pituitary tumors *in vitro* and *in vivo* ^81^. Whether USP8 has similar HIF-1–stabilizing properties and thus influences angiogenic growth factor production in corticotroph tumors needs to be clarified.

The intratumoral formation of new vessels requires the disruption of cell–cell contacts between tumor cells. Thus, during neovascularization, not only factors stimulating the growth of vessels but also factors degrading the extracellular matrix of tumor cells are produced ^76^. In these processes, enzymes of the matrix metalloproteinase (MMP) family and their negative regulators, the tissue inhibitors of MMPs (TIMPs), play important roles ^82, 83^, but numerous other factors are also involved ^76^. These soluble factors not only may locally act to pave the way for invading vessels but may diffuse into the tumor cell matrix and into the anatomical structures surrounding the adenomas. Therefore, the process of neovascularization bears the risk that matrix degrading components support the migration of tumor cells to invade surrounding tissues at the edge of the tumors ^84, 85^. The link between angiogenesis and invasiveness is supported by the observation that RSUME not only has angiogenic properties in corticotroph tumors but also stimulates migration and invasive growth of corticotroph tumor cells ^86^. Normally, corticotroph macroadenomas show invasive growth characteristics, but in Crooke’s cell adenomas, for instance, invasive growth has already been observed in microadenomas ^2^.

Comparative studies between the normal anterior pituitary and CAs have identified hundreds of genes, miRs, proteins, and peptides that are up- or down-regulated in the adenomas ^87^. This is not surprising as, in the adult anterior pituitary, cells normally do not grow and neither angiogenic nor invasive processes take place. Thus, all factors related to these events are somehow differentially expressed in the corticotropinomas, but it should be emphasized that these aberrant expression patterns are mainly a consequence, not a cause, of CAs. Correspondingly, there are also differences between non-invasive and invasive growing corticotroph tumors ^88, 89^, such as differential expression of the miR106b~25 cluster, the cyclin D2 ( *CCND2*), zinc finger protein 676 ( *ZNF676*), death-associated protein kinase 1 ( *DAPK1*), and *TIMP2* genes as well as genes associated with TGF-β and G protein signaling pathways, DNA damage response pathways, focal adhesion–associated pathways, and others ^48, 50, 88– 92^. Although it has been speculated that all of the aberrantly expressed factors play a role in corticotropinoma expansion and thus represent putative targets for pharmacological treatment, this has been proven *in vivo* in mouse allograft corticotroph tumor models in only a few cases but has not led to corresponding clinical studies.

### Corticotroph carcinomas

According to the 2017 WHO classification, pituitary carcinomas, which represent less than 0.1% of all pituitary tumors, are characterized by their ability to form metastases and both hereditary and sporadic pituitary tumors can transform into carcinomas ^2^. Although the proportion of CAs within all pituitary tumor types is relatively small, they represent about 35% to 48% of all pituitary carcinomas ^6, 7, 93^. This may be a consequence of the relative high proportion of aggressive tumor types (Crooke’s cell adenomas, Nelson’s tumors, and silent CAs) among the corticotroph tumors ^2^. In most reports, several common features of corticotroph carcinomas are evident, such as repeated recurrence of the adenomas after incomplete surgical resection and transient response but finally resistance to different pharmacological therapies or to radiotherapy ^6, 7, 66, 68, 73, 74, 94^. Usually, decades separate the first diagnosis of the CA and the full expression of the carcinoma phenotype. At present, there is no evidence of a specific genetic or epigenetic background or a common factor that is responsible for the development of pituitary carcinomas ^95^. In a recent meta-analysis, in which pituitary carcinomas were compared with aggressive adenomas, a number of factors related to the stimulation of cell growth, angiogenesis, and invasiveness (cyclin D1, VEGF, MMP9, miR-122, and miR-493) were found to be up-regulated, whereas several factors, including growth inhibitors and apoptosis inducers (p16Ink4A, p27Kip1, MT3, BCL-2, Bax, Bcl-X, and O ^6^-methylguanine DNA methyltransferase [MGMT]), were down-regulated ^95, 96^. However, these factors are also differently expressed in invasive versus non-invasive adenomas, in micro- versus macro-adenomas, or in poorly versus densely vascularised pituitary tumors ^76, 84, 87^. These data suggest that an accumulation of changes associated with increased pituitary tumor aggressiveness rather than a carcinoma-specific mutation or epigenetic change (or both) will finally lead to development of metastasizing pituitary carcinomas ^93, 95^.

Temozolomide, an alkylating chemotherapeutic drug, has been identified as an effective compound in the treatment of pituitary carcinomas ^7, 94^. The effect of temozolomide is reduced in pituitary tumors with high expression of MGMT. Crooke’s cell adenomas, Nelson’s tumors, invasively growing corticotroph macroadenomas, and corticotroph carcinomas mostly have low MGMT expression ^2, 97– 100^. This may explain the relatively good response of corticotroph carcinomas to temozolomide therapy in several cases ^7, 94^. However, this also suggests that advanced aggressive corticotropinoma types may already be treated with temozolomide to prevent transformation into corticotroph carcinomas ^101^.

## Conclusions

CAs are mostly sporadic tumors; only a small proportion of CAs have a hereditary background and are associated with neuroendocrine syndromes or are FIPAs. In sporadic CAs, a recurrent activating mutation of the *USP8* gene, which is present in about 40% of ACTH-secreting CAs, has recently been found. The USP8 mutation participates in CA pathophysiology by stimulating EGFR expression and thus enhances EGF-induced ACTH production. As mutated USP8 also increases SSTR5 expression, CAs with USP8 mutation respond better to treatment with the somatostatin analogue pasireotide which acts through SSTR5. No other recurrent mutations have been identified in CAs, suggesting that single different mutations or epigenetic changes (or both) may play a role in CA initiation. The ACTH secretion by corticotroph tumor cells, in contrast to normal corticotroph cells, is resistant to inhibitory glucocorticoid feedback control, and HSP90 overexpression has recently been shown to be involved in Cushing’s disease. Thus, HSP90 inhibitors like silibinin may be promising drugs to restore glucocorticoid responsiveness of CAs and to reverse the pathophysiological changes of Cushing’s disease. During the progression of CAs from microadenomas to aggressive macroadenomas and finally rare cases of corticotroph carcinomas, multiple factors (genes, miRs, proteins, and peptides) are up- or down-regulated and thus may provide putative targets for new pharmacological treatment strategies. However, despite remarkable progress in understanding corticotroph tumor initiation and progression, a lot of work is still needed to explore the underlying mechanisms, and the translation of new findings into clinical applications for diagnosis and therapy of corticotropinomas is still unsatisfactory.

## Abbreviations

ACTH, adrenocorticotropic hormone; AIP, aryl hydrocarbon receptor-interacting protein; CA, corticotroph adenoma; CABLES1, Cdk5 and ABL enzyme substrate 1; CNC, Carney complex; EGF, epidermal growth factor; EGFR, epidermal growth factor receptor; ERK, extracellular signal-regulated kinase; FIPA, familial isolated pituitary adenomas; GR, glucocorticoid receptor; HIF-1, hypoxia-inducible factor 1; HSP90, heat shock protein 90; MAPK, mitogen-activated protein kinase; MEN, multiple endocrine neoplasia; MGMT, O ^6^-methylguanine DNA methyltransferase; miR, microRNA; MMP, matrix metalloproteinase; NFPA, non-functioning pituitary adenoma; POMC, proopiomelanocortin; Rb, retinoblastoma; RSUME, RWD-domain-containing sumoylation enhancer; SSTR5, somatostatin receptor type 5; TGF, transforming growth factor; TIMP, tissue inhibitor of metalloproteinase; TR4, testicular orphan nuclear receptor 4; USP8, ubiquitin-specific protease 8; VEGF-A, vascular endothelial growth factor-A; WHO, World Health Organization.
